# Effects of balanced solution on short-term outcomes in traumatic brain injury patients: a secondary analysis of the BaSICS randomized trial

**DOI:** 10.5935/0103-507X.20220261-en

**Published:** 2022

**Authors:** Fernando Godinho Zampieri, Lucas Petri Damiani, Rodrigo Santos Biondi, Flávio Geraldo Rezende Freitas, Viviane Cordeiro Veiga, Rodrigo Cruvinel Figueiredo, Ary Serpa-Neto, Airton Leonardo de Oliveira Manoel, Tamiris Abait Miranda, Thiago Domingos Corrêa, Luciano César Pontes de Azevedo, Nilton Brandão da Silva, Flavia Ribeiro Machado, Alexandre Biasi Cavalcanti, BRICNet

**Affiliations:** 1 Research Institute, HCor-Hospital do Coração - São Paulo (SP), Brazil.; 2 Brazilian Research in Intensive Care Network (BRICNet) - São Paulo (SP), Brazil.; 3 Instituto de Cardiologia do Distrito Federal - Brasília (DF), Brazil.; 4 Department of Anesthesiology, Pain and Intensive Care, Universidade Federal de São Paulo - São Paulo (SP), Brazil.; 5 BP-A Beneficência Portuguesa de São Paulo - São Paulo (SP), Brazil.; 6 Hospital Maternidade São José, Centro Universitário do Espírito Santo - Colatina (ES), Brazil.; 7 Hospital Israelita Albert Einstein - São Paulo (SP), Brazil.; 8 Hospital Paulistano - São Paulo (SP), Brazil.; 9 Hospital Sírio-Libanês - São Paulo (SP), Brazil.; 10 Faculdade de Medicina, Universidade Federal de Ciências Médicas - Porto Alegre (RS), Brazil.

**Keywords:** Balanced solutions, Saline solution, Brain injuries, traumatic, Hospital mortality, Mortality, Critical care

## Abstract

**Objective:**

To describe the effects of balanced solution use on the short-term outcomes of patients with traumatic brain injury enrolled in BaSICS trial.

**Methods:**

Patients were randomized to receive either 0.9% saline or balanced solution during their intensive care unit stay. The primary endpoint was 90-day mortality, and the secondary outcomes were days alive and free of intensive care unit stay at 28 days. The primary endpoint was assessed using Bayesian logistic regression. The secondary endpoint was assessed using a Bayesian zero-inflated beta binomial regression.

**Results:**

We included 483 patients (236 in the 0.9% saline arm and 247 in the balanced solution arm). A total of 338 patients (70%) with a Glasgow coma scale score ≤ 12 were enrolled. The overall probability that balanced solutions were associated with higher 90-day mortality was 0.98 (OR 1.48; 95%CrI 1.04 - 2.09); this mortality increment was particularly noticeable in patients with a Glasgow coma scale score below 6 at enrollment (probability of harm of 0.99). Balanced solutions were associated with -1.64 days alive and free of intensive care unit at 28 days (95%CrI -3.32 - 0.00) with a probability of harm of 0.97.

**Conclusion:**

There was a high probability that balanced solutions were associated with high 90-day mortality and fewer days alive and free of intensive care units at 28 days.

**ClinicalTrials.gov:** NCT02875873

## INTRODUCTION

Balanced solutions have been suggested to be beneficial for critically ill patients, although their precise role is presently unclear.^(1)^ Among the four largest trials conducted assessing balanced solutions in a mixed population of critically ill patients,^(2-5)^ only one met the predefined criteria for “statistical significance” (p < 0.05) for a composite endpoint for mortality, need for kidney replacement therapy (KRT) or doubling of creatinine, with the remaining trials yielding neutral results.^(3)^

Traumatic brain injury (TBI) is a special population with some particularities in fluid management. It has been suggested that balanced solutions could exert harmful effects in this population, which could be related to the osmolarity of the fluid infused.^(6,7)^ In the BaSICS (Balanced Solution in Intensive Care Study), patients were randomized to receive either 0.9% saline or Plasma-Lyte 148 (an isotonic solution with lower chloride content) for maintenance, dilution, and fluid challenges during intensive care unit (ICU) stay.^(4)^ Although BaSICS reported neutral results, it suggested a possible harm of balanced solutions in the prespecified subgroup of patients with TBI despite the use of an isotonic fluid in the intervention arm.^(4)^

We aimed to further evaluate the subgroup of patients with TBI in the BaSICS trial, describing the effect of balanced solutions in this population on mortality (our primary endpoint), days alive and free of ICU, and organ dysfunctions. We hypothesized that balanced solutions would be harmful in this subgroup, with an interaction between neurological status at study enrollment, as assessed by the neurological component of the Sequential Organ Failure Assessment (SOFA) score,^(6)^ and the intervention.

## METHODS

### Study design and patients

A secondary analysis of the BaSICS trial based on a prespecified subgroup. All patients with complete primary endpoint information and with a registered diagnosis of TBI at baseline in the case report form were included in the analysis.

### Procedures

Details on the inclusion and exclusion criteria in the BaSICS trial can be found in the main publication^(4)^ and protocol.^(8)^ In brief, patients requiring at least one fluid challenge with risk factors for acute kidney injury and without a discharge plan in the next 24 hours were considered for inclusion in the trial. The study assessed both the effects of fluid type and two different infusion rates for fluid bolus; this secondary analysis considers only the fluid type analysis.

### Measurements

Due to its pragmatic design, information on TBI was collected as a yes/no question, without further data on the trauma mechanism or presentation. We collected information on the primary outcome (90-day survival), as well as fluid use and organ dysfunctions (as measured by SOFA score) on specific days.^(9)^ The neurological component of the SOFA score was estimated based on a physical exam for nonsedated patients or the last known Glasgow coma scale (GCS) value for patients who were sedated (values of neurological SOFA of 0 to 4 are equivalents to GCS of 15, 13 - 14, 10 - 12, 6 - 9, and < 6, respectively).^(9)^

### Endpoints

The primary endpoint was 90-day mortality. The key secondary endpoint was days alive and free of ICU stay at 28 days. Exploratory endpoints included the need for KRT at 90 days and organ dysfunctions (neurological, cardiovascular, and renal organ dysfunctions - all measured using SOFA score) at Day 3 after enrollment.

### Statistical analysis

The primary endpoint was assessed using a Bayesian hierarchical logistic regression model with intervention as a predictor and enrolling site as a random effect. Alternatively, the model was adjusted for baseline neurological SOFA score (as factor, from 0 to 4), the intervention and their interaction as predictors, with enrolling site also considered a random effect. The full model’s syntax is provided in appendix 1S (Supplementary material). A neutral normal prior centered at an odds ratio (OR) of 1 (log[OR] = 0) and with standard deviation of 0.355 was applied for all fixed predictors in the analysis; this prior has 0.95 of its probability mass for OR between 0.5 and 2 (neutral, moderate strength prior).^(10)^ Results are expressed in terms of OR or, for the alternative model, as conditional effects OR for the intervention at neurological SOFA score. We provide the posterior distribution for the OR (in log scale), the 95% credible interval (95%CrI), and the probability of direction (that is, the probability that OR > 1.0). Equivalence testing was made by arbitrarily defining an equivalence margin equal to an OR between 1.1 and 1/1.1.^(10)^

Days alive and free of ICU stay were modeled following a zero inflated beta binomial model. We performed both an unadjusted and an adjusted analysis. The adjusted analysis was adjusted for baseline neurological SOFA, intervention, and their interaction as predictors. Patients who died up to 28 days received a value of zero. We obtained the difference in days alive and free of ICU through sampling the expected probability distribution of days alive and free of ICU from the model and summarizing it as median, 95%CrI, the probability the intervention is associated with fewer days alive and free of ICU, and probability that the difference is within a margin of one day (equivalence margin). The code syntax for the secondary endpoint is also shown in appendix 1S (Supplementary material).

Need for KRT was assessed although a Bayesian logistic model adjusted for intervention, total SOFA score and their interaction with neutral priors, with results presented as median OR, 95%CrI, probability of direction, and probability of equivalence. Other exploratory endpoints were modeled using a cumulative ordinal Bayesian model for neurological, cardiovascular, and renal components of the SOFA score. Models were adjusted for the respective baseline SOFA component value, intervention, and their interaction. The endpoint was coded so it considered the five levels of each SOFA component plus attributing early discharges up to Day 3 and early mortality for patients who died on up to Day 3. The results are presented as OR and 95%CrI for a worse value in the scale under a cumulative logit model. We applied the same margin of equivalence as for the primary endpoint.

All analyses were performed using R software version 4.2.0^(11)^ using brms,^(12)^ and tidybayes^(13)^ with ggplot2 for visualization.^(14)^

## RESULTS

**Figure 1:**
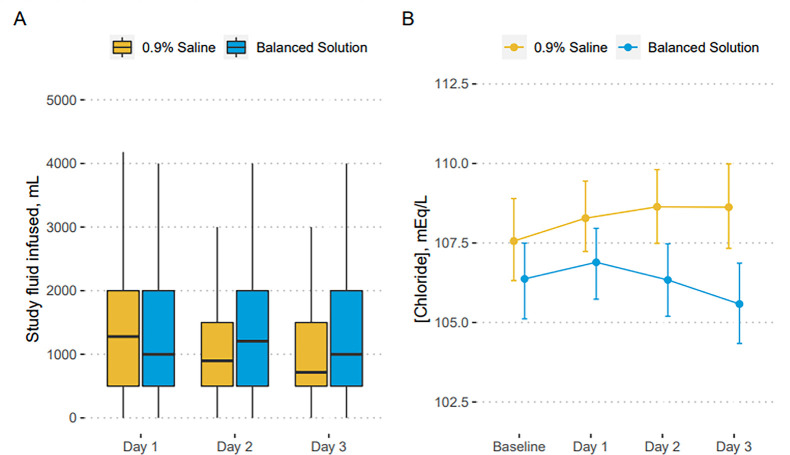
(A) Fluid use during the first three days in the trial; (B) Chloride values for patients who had chloride measured at baseline and the following 3 days, stratified by group. Error bars represent 95% confidence intervals obtained through nonparametric bootstrapping.

**Figure 2:**
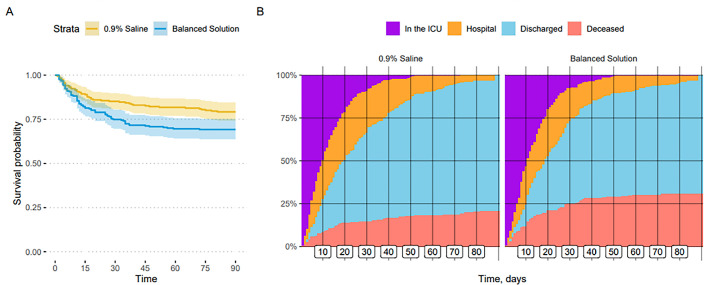
(A) Survival curves for included patients, stratified according to intervention arm. (B) Patient status over time up to 90 days (only the first intensive care unit admission is considered); each vertical line represents 10-day intervals.

Of the 10,520 patients, 10,490 had full information on the reason for admission; of those, 483 had a diagnosis of TBI at admission and were included (236 in the 0.9% saline arm and 247 in the balanced solution arm). Patient features and unadjusted outcomes are shown in table 1. Most patients were male, with a median age of 44 years. A total of 338 patients (70%) were enrolled with a GCS score ≤ 12 (as defined by a neurologic SOFA score > 2). The overall mortality at 90 days was 26% (21% in the 0.9% saline group and 31% in the balanced solution group). Patients received an average of 2,430mL of study fluid (interquartile range - IQR 1,250 - 4,500mL) during the first three days after enrollment (2,381mL [IQR 1,191 - 4,000] in the 0.9% saline group and 2,487mL [IQR 1,404 - 4,976] in the balanced solution group). Fluid use during the first three days is shown in figure 1A, and serum chloride levels for patients who had their chloride measured are shown in figure 1B. A Kaplan‒Meier plot for survival stratified according to randomization arm is shown in figure 2A, and patient status over time up to 90 days is shown in figure 2B.

**Table 1:** Baseline features and outcomes of included patients according to treatment group

	0.9% saline solution n = 236	Balanced solution n = 247
Characteristic		
Age	43 [28 - 61]	45 [30 - 63]
Male sex	183 (78)	203 (82)
APACHE II	15 [10 - 20]	15 [11 -20]
SOFA	7.0 [4.0 - 9.0]	7.0 [5.0 - 10.0]
Neurological SOFA		
0 (GCS 15)	40 (17)	33 (13)
1 (GCS 13 - 14)	40 (17)	32 (13)
2 (GCS 10 - 12)	27 (11)	30 (12)
3 (GCS 6 - 9)	47 (20)	84 (34)
4 (GCS < 6)	82 (35)	68 (28)
Cardiovascular SOFA		
0 (no hypotension)	87 (37)	94 (38)
1 (MAP < 70mmHg)	34 (14)	28 (11)
2 (dopamine < 5mcg/kg/min) or dobutamine)	0 (0)	2 (0.8)
3 (norepinephrine < 0.1mcg/kg/min)	30 (13)	39 (16)
4 (norepinephrine > 0.1mcg/kg/min)	85 (36)	84 (34)
Hypotension or vasopressor use at enrollment	133 (56)	148 (60)
Mechanical ventilation at enrollment	158 (67)	173 (70)
Outcomes		
ICU mortality	33 (14)	52 (21)
Hospital mortality	42 (18)	73 (30)
90-day mortality	49 (21)	76 (31)
Need for KRT	18 (7.6)	22 (8.9)
ICU length-of-stay (dias)	9 [4 - 18]	10 [6 - 18]
Days alive and free of ICU at 28 days	16 [4 - 23]	13 [0 -21]
Hospital length-of-stay (days)	19 [9 - 37]	18 [10 - 32]

APACHE II - Acute Physiology and Chronic Health Evaluation II; SOFA - Sequential Organ Failure Assessment Score; GCS - Glasgow coma scale; MAP - mean arterial pressure; ICU - intensive care unit; KRT - kidney replacement therapy. The results are expressed as the median [interquartile range] or n (%).

ICU - intensive care unit.

**Figure 3:**
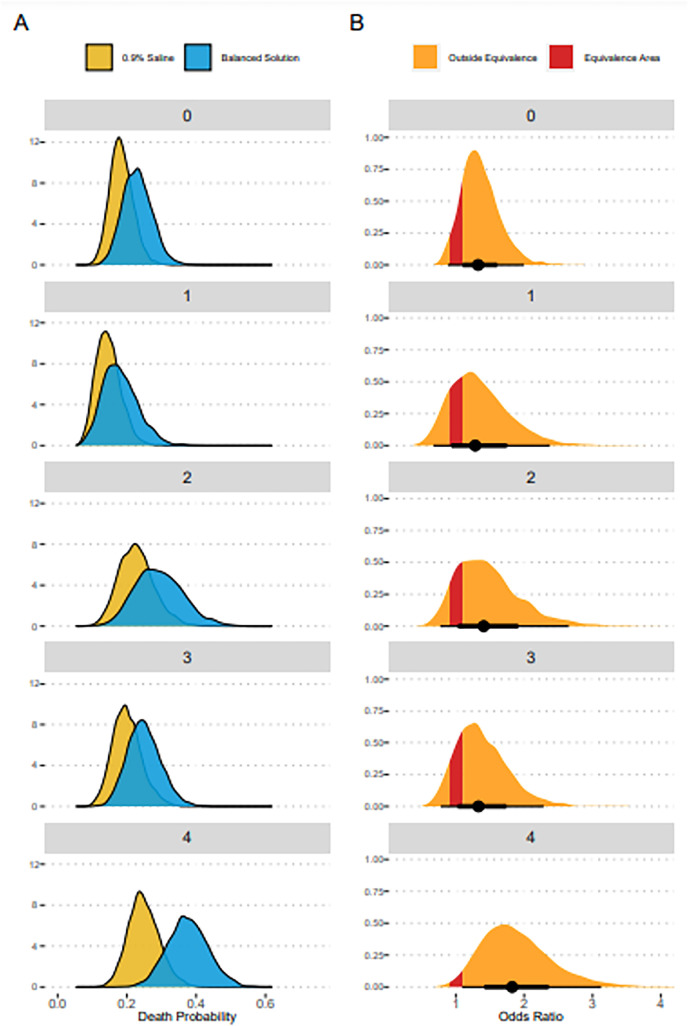
(A) Expected posterior predicted mortality according to baseline neurological Sequential Organ Failure Assessment component and intervention. (B) The respective odds ratios obtained from the probabilities shown in panel (A), with a region of equivalence highlighted.

The results for the primary endpoint are shown in figure 1S (Supplementary material), figure 3, and table 2. Figure 1S (Supplementary material) shows the posterior odds distribution for the unadjusted 90-day mortality analysis. Figure 3A shows the expected posterior predictions for the adjusted 90-day mortality for both the 0.9% saline and balanced solutions groups according to the neurological SOFA score at enrollment, and figure 3B shows the distribution of the OR for each neurological SOFA score at enrollment. Numerical summaries of effect sizes are shown in table 2. The overall probability that balanced solutions were associated with higher 90-day mortality in the unadjusted analysis was 0.982. Under the adjusted analysis, this mortality increment was particularly noticeable in patients with a neurological SOFA score of 4 (corresponding to a GCS score < 6).

**Table 2:** Summary of the effect sizes for primary and secondary endpoints-

	Absolute difference Median (95%CrI)	Odds ratio Median (95%CrI)	Probability of harm^*^	Probability of equivalence†
90-day mortality - unadjusted model				
All patients	0.07 (0.01 - 0.14)	1.48 (1.04 - 2.09)	0.98	0.05
90-day mortality - adjusted model				
Neurological SOFA 0 (GCS 15)	0.04 (-0.02 - 0.12)	1.32 (0.88 - 1.99)	0.92	0.14
Neurological SOFA 1 (GCS 13 - 14)	0.03 (-0.05 - 0.12)	1.28 (0.67; 2.37)	0.77	0.16
Neurological SOFA 2 (GCS 10 - 12)	0.06 (-0.05 - 0.19)	1.40 (0.77; 2.64)	0.86	0.15
Neurological SOFA 3 (GCS 6 - 9)	0.05 (-0.04 - 0.14)	1.33 (0.78 - 2.28)	0.85	0.16
Neurological SOFA 4 (GCS < 6)	0.12 (0.02 - 0.24)	1.82 (1.09 - 3.12)	0.99	0.02
Need for KRT at 90 days				
All patients	0.01 (-0.02 - 0.05)	1.20 (0.70 - 2.09)	0.73	0.21
Days alive and free of ICU - unadjusted model				
	-1.64 (-3.32 - 0.00)	-	0.97	0.23
Days alive and free of ICU - adjusted model				
Neurological SOFA 0 (GCS 15)	-0.82 (-4.24 - 2.45)	-	0.69	0.40
Neurological SOFA 1 (GCS 13 - 14)	0.68 (-3.58 - 4.74)	-	0.37	0.33
Neurological SOFA 2 (GCS 10 - 12)	-1.20 (-5.54 - 3.70)		0.70	0.30
Neurological SOFA 3 (GCS 6 - 9)	-1.80 (-4.67 - 1.17)	-	0.89	0.27
Neurological SOFA 4 (GCS < 6)	-3.19 (-5.75 - -0.412)	-	0.99	0.06

95%CrI - 95% credible interval; SOFA - Sequential Organ Failure Assessment Score; GCS - Glasgow coma scale; ICU - intensive care unit.

* Probability that OR > 1.0 for mortality (primary endpoint) or that intervention is associated with fewer days alive and free of intensive care unit (secondary endpoint); † Probability that odds ratio is between 1/1.1 to 1.1 for mortality (primary endpoint) or that intervention effect on days alive and free of intensive care unit (secondary endpoint) is within a one-day margin.

The results for KRT were inconclusive (Table 2); the probability of harm was close to 0.73, and credible intervals were wide. On unadjusted analysis, balanced solutions were associated with -1.64 days alive and free of ICU (95%CrI -3.32 - 0.00); overall probability of harm 0.97. Consistent with the primary endpoint, the probability of harm was higher for higher neurological SOFA scores at enrollment, peaking over 0.99 for neurological SOFA scores of 4 (Table 2, Figure 2S - Supplementary material).

Other exploratory endpoints are shown in table 1S and figures 3S and 4S (Supplementary material). The probability of transitioning to a higher neurological component SOFA score or death was high for balanced solutions when the baseline neurological SOFA was 4. There was no clear signal of higher odds of transitioning to a higher SOFA component value or death for either cardiovascular or renal SOFA components.

## DISCUSSION

In this secondary *post hoc* analysis of the BaSICS trial, we found an above 0.95 probability that being enrolled to receive balanced solutions was associated with increased 90-day mortality in patients with TBI. The probability of harm was particularly high in patients with higher neurological SOFA scores at enrollment, peaking at 0.99 in patients with neurological SOFA scores of 4 (GCS below 6 points). Balanced solutions may also decrease the number of days alive and free of ICU stay in this population, with a very high probability of reducing the number of days alive and free of ICU stay by at least one day for patients with high baseline neurological SOFA scores. We also found that balanced solutions may be associated with progression to ordinal status of neurological SOFA or death in patients with higher baseline neurological SOFA, while the results were inconclusive for cardiovascular and renal SOFA. The results for the need for KRT were also inconclusive, and we were unable to confirm the important effects of balanced solution at this endpoint in TBI patients.

Our findings should be interpreted in the context of a secondary exploratory analysis of a large trial, considering that our sample is limited, and the results suffer from imprecision. However, our results are aligned with trends observed in the SMART trial, where point estimates favored 0.9% saline over balanced solutions in TBI patients,^(3)^ and in other neurological conditions, such as subarachnoid hemorrhage.^(15)^ Our results are also aligned with the main subgroup report of the BaSICS trial. Due to the use of neutral priors and different model specifications, the result in this analysis is more conservative than the raw report in the TBI subgroup in the main trial. We further extended the trial original subgroup analysis by applying a different methodology (Bayesian model) considering important factors known to be related to outcome in TBI patients, including age^(16)^ and a proxy of GCS; our results suggest not only an impact at 90-day mortality but also an immediate association between balanced solution use and mortality, which can be seen in both the survival curves (Figure 2) and in the days alive and free of ICU analyses. We also provide evidence that possible harm of balanced solutions is more pronounced in more severe patients (suggesting a gradient of effect, which enhances the association probability), that is, those randomized with a lower GCS value; this may be related to increased harm in patients with more severe TBI (who carry an increased risk of complications and higher intracranial pressure), or consequence of the higher number of events in this group (which increases precision of estimates). It should be highlighted, however, that for all subgroups of neurological SOFA, the expected probability distribution of mortality was somewhat higher in the balanced solution group. It is important to highlight that despite a chloride gradient between groups with lower values in balanced solutions, we found higher mortality in TBI patients and neutral, inconclusive results for KRT, which may be an important endpoint for trials assessing fluid type in critically ill patients.

Concerns over the safety of balanced solutions in TBI patients were pressing enough so that the PLUS trial,^(5)^ another large, individually randomized controlled trial assessing the possible effects of balanced solutions, was chosen to exclude TBI patients from inception. Reasons for the possible harm of balanced solutions in TBI patients are uncertain. Plasma-Lyte 148 ®, an isotonic solution, was used in BaSICS, which may partially avoid issues of low tonicity solutions in increasing intracranial pressure.^(17)^ The effects of the buffer anion of balanced solutions in traumatic brain injury, however, are presently unclear. Plasma-Lyte 148® uses acetate as the primary buffer, which has largely unknown effects on brain circulation at isotonic levels.^(17)^ Acetate may exert important cardiovascular functions, including vasodilation;^(18)^ in the main BaSICS trial, balanced solution was associated with more cardiovascular SOFA values above 2 on the third day after enrollment, which may be related to buffer effects in the circulation.^(4)^ Finally, differences in sodium concentration, another major determinant of plasmatic oncotic pressure, are different between Plasma-Lyte 148 and 0.9% saline, which may explain part of the results. In the lack of true mechanistic data, this remains speculative.

Clinical trials should be performed to assess possible benefit and not to exclude harm, except in doubtful scenarios of an intervention that may present with significant heterogeneity of treatment effect. For example, if the results of any of the large trials on balanced solutions clearly suggested an overall benefit of relevant patient-centered outcomes (mortality, length of stay, among others) but had dubious findings in TBI, it would be reasonable to confirm or refute this finding in a dedicated trial. However, the benefit of balanced solutions, if any, appears to be small,^(1)^ related to fluid use before enrollment,^(19)^ and unclear for long-term outcomes.^(1)^ Regarding TBI patients, when faced with a strong signal for harm for this type of fluid in this population, clinicians may choose to avoid balanced solutions. An individual patient metanalysis of the large trials of balanced solutions may clarify the trends observed in this analysis.^(20)^

This manuscript has several limitations. It is a *post hoc* analysis of a randomized controlled trial; therefore, although the subgroup was prespecified in the main analysis, it should be seen as exploratory. We had no data on the mechanisms of trauma or type of brain injury each patient had; it is conceivable that effects could vary according to the type of neurological injury (for example, extradural hematoma versus diffuse brain swelling). There is also no information on how patients were managed, including sedation use and intracranial pressure monitoring; therefore, we are unable to provide a mechanistic hypothesis for our findings. Additionally, we lack information on sodium levels, which are also a major determinant of oncotic pressure. Finally, mortality is not the sole outcome of importance for TBI patients, and we lacked data on long-term neurological outcomes.^(21)^

## CONCLUSION

There is a high probability that balanced solutions may be associated with increased mortality in critically ill patients with traumatic brain injury. This association is more pronounced in patients with high neurological impairment at enrollment. Given the unclear benefits of balanced solutions in critically ill patients, it is reasonable to avoid balanced solutions in this specific subgroup.

## References

[1] Hammond NE, Zampieri FG, Di Tanna GL, Garside T, Adigbli D, Cavalcanti AB (2022). balanced crystalloids versus saline in critically ill adults - a
systematic review with meta-analysis. NEJM Evid.

[2] Young P, Bailey M, Beasley R, Henderson S, Mackle D, McArthur C, McGuinness S, Mehrtens J, Myburgh J, Psirides A, Reddy S, Bellomo R, SPLIT Investigators, ANZICS CTG (2015). Effect of a buffered crystalloid solution vs saline on acute
kidney injury among patients in the intensive care unit: the SPLIT
randomized clinical trial. JAMA.

[3] Semler MW, Self WH, Wanderer JP, Ehrenfeld JM, Wang L, Byrne DW, Stollings JL, Kumar AB, Hughes CG, Hernandez A, Guillamondegui OD, May AK, Weavind L, Casey JD, Siew ED, Shaw AD, Bernard GR, Rice TW, SMART Investigators and the Pragmatic Critical Care Research
Group (2018). Balanced crystalloids versus saline in critically ill
adults. N Engl J Med.

[4] Zampieri FG, Machado FR, Biondi RS, Freitas FG, Veiga VC, Figueiredo RC, Lovato WJ, Amêndola CP, Serpa-Neto A, Paranhos JL, Guedes MA, Lúcio EA, Oliveira-Júnior LC, Lisboa TC, Lacerda FH, Maia IS, Grion CMC, Assunção MS, Manoel AL, Silva-Junior JM, Duarte P, Soares RM, Miranda TA, de Lima LM, Gurgel RM, Paisani DM, Corrêa TD, Azevedo LC, Kellum JA, Damiani LP, Brandão da Silva N, Cavalcanti AB, BaSICS investigators and the BRICNet members (2021). Effect of intravenous fluid treatment with a balanced solution vs
0.9% saline solution on mortality in critically ill patients: the BaSICS
randomized clinical trial. JAMA.

[5] Finfer S, Micallef S, Hammond N, Navarra L, Bellomo R, Billot L, Delaney A, Gallagher M, Gattas D, Li Q, Mackle D, Mysore J, Saxena M, Taylor C, Young P, Myburgh J, PLUS Study Investigators (2022). Australian New Zealand Intensive Care Society Clinical Trials
Group. Balanced multielectrolyte solution versus saline in critically ill
adults. N Engl J Med.

[6] Rowell SE, Fair KA, Barbosa RR, Watters JM, Bulger EM, Holcomb JB (2016). The impact of pre-hospital administration of lactated ringer’s
solution versus normal saline in patients with traumatic brain
injury. J Neurotrauma.

[7] Roquilly A, Loutrel O, Cinotti R, Rosenczweig E, Flet L, Mahe PJ (2013). Balanced versus chloride-rich solutions for fluid resuscitation
in brain-injured patients: a randomised double-blind pilot
study. Crit Care.

[8] Zampieri FG, Azevedo LC, Corrêa TD, Falavigna M, Machado FR, Assunção MS, Lobo SM, Dourado LK, Berwanger O, Kellum JA, Brandão N, Cavalcanti AB, BaSICS Investigators and the BRICNet (2017). Study protocol for the Balanced Solution versus Saline in
Intensive Care Study (BaSICS): a factorial randomised trial. Crit Care Resusc.

[9] Vincent JL, Moreno R, Takala J, Willatts S, De Mendonça A, Bruining H (1996). The SOFA (Sepsis-related Organ Failure Assessment) score to
describe organ dysfunction/failure. On behalf of the Working Group on
Sepsis-Related Problems of the European Society of Intensive Care
Medicine. Intensive Care Med.

[10] Zampieri FG, Casey JD, Shankar-Hari M, Harrell FE Jr, Harhay MO (2021). Using Bayesian methods to augment the interpretation of critical
care trials. An overview of theory and example reanalysis of the alveolar
recruitment for acute respiratory distress syndrome trial. Am J Respir Crit Care Med.

[11] R Core Team (2022). R: A language and environment for statistical computing.

[12] Bürkner PC (2017). brms: An R Package for Bayesian Multilevel Models Using
Stan. J Stat Softw.

[13] Kay M (2021). tidybayes: Tidy Data and Geoms for Bayesian Models. R package version
3.0.2.

[14] Wickham H (2016). ggplot2: Elegant Graphics for Data Analysis.

[15] Mistry MA, Magarik JA, Feldman MJ, Wang L, Lindsell CJ, Fusco MR (2022). Saline versus balanced crystalloids for adults with aneurysmal
subarachnoid hemorrhage: a subgroup analysis of the SMART
trial. Stroke Vasc Interv Neurol.

[16] Salottolo K, Panchal R, Madayag RM, Dhakal L, Rosenberg W, Banton KL (2021). Incorporating age improves the Glasgow Coma Scale score for
predicting mortality from traumatic brain injury. Trauma Surg Acute Care Open.

[17] Weinberg L, Collins N, Van Mourik K, Tan C, Bellomo R (2016). Plasma-Lyte 148: a clinical review. World J Crit Care Med.

[18] Aizawa Y, Shibata A, Ohmori T, Kamimura A, Takahashi S, Hirasawa Y (1978). Hemodynamic effects of acetate in man. J Dial.

[19] Zampieri FG, Machado FR, Biondi RS, Freitas FG, Veiga VC, Figueiredo RC (2022). Association between type of fluid received prior to enrollment,
type of admission, and effect of balanced crystalloid in critically ill
adults: a secondary exploratory analysis of the BaSICS clinical
trial. Am J Respir Crit Care Med.

[20] Zampieri FG, Cavalcanti AB, Di Tanna GL, Damiani LP, Hammond NE, Machado FR (2022). Protocol for balanced versus saline trialists: living systematic
review and individual patient data meta-analysis of randomised controlled
trials (BEST-Living study). Crit Care Resusc.

[21] Maas AI, Marmarou A, Murray GD, Teasdale SG, Steyerberg EW (2007). Prognosis and clinical trial design in traumatic brain injury:
the IMPACT study. J Neurotrauma.

