# Supplementary material for: Effects of balanced solution on short-term outcomes in traumatic brain injury patients: a secondary analysis of the BaSICS randomized trial
Source: Rev Bras Ter Intensiva. 2022 Oct-Dec;34(4):410–7. doi: 10.5935/0103-507X.20220261-en (PMC9987002; doi:10.5935/0103-507X.20220261-en)
Supplement: Supplementary file 1 [file rbti-34-04-0410-suppl1.pdf]

Effects of balanced solution on short-term outcomes in traumatic brain injury patients: a secondary analysis of the BaSICS randomized trial

Efeitos da solução balanceada em desfechos de curto prazo em pacientes com traumatismo craniencefálico: uma análise secundária do ensaio clínico randomizado BaSICS

Fernando Godinho Zampieri[1,2], Lucas Petri Damiani[1], Rodrigo Santos Biondi[3], Flávio Geraldo Rezende Freitas[4], Viviane Cordeiro Veiga[2,5], Rodrigo Cruvinel Figueiredo[6], Ary Serpa-Neto[7], Airton Leonardo de Oliveira Manoel[8], Tamiris Abait Miranda[1], Thiago Domingos Corrêa[7], Luciano César Pontes de Azevedo[2,9], Nilton Brandão da Silva[10], Flavia Ribeiro Machado[2,4], Alexandre Biasi Cavalcanti[1,2] and the BRICNet

Appendix 1S - Code snippets.

Code for primary analysis

The primary analysis was made following two different models, one unadjusted and other adjusted model for baseline neurological Sequential Organ Failure Assessment (SOFA). The model for the unadjusted analysis was a simple model, while the adjusted model considered age and the baseline neurological SOFA, intervention and their interaction. Models were run with four chains and using the neutral moderate strength prior. Results were summarized as differences in probability and odds ratios, coupled with an equivalence analysis of percentage of posterior that was contained in a region of equivalence defined as an odds ratio between 1/1.1 and 1.1. “csofan” stands for baseline neurological SOFA score at enrollment (factor, from 0 to 4). We used {brms}, {tidybayes}, {emmeans}, and {dplyr} for this analysis. {cmdstanr} was used as a backend for the interface between R and Stan. We used R version 4.2.0.

```
#### PRIMARY ANALYSIS MODELS
#Set prior
nprior <- prior(normal(0, 0.355), class = “b”))
#Run Models
## Unadjusted
m1_simple<-brm(death ~ intervention + (1|hospital), data = maindata, family = “bernoulli”, prior = nprior, backend = “cmdstan”, seed=123, chains = 4, cores = 4)
## Adjusted
m1<-brm(death ~ age + csofan*intervention + (1|hospital), data = maindata, family = “bernoulli”, prior = nprior, backend = “cmdstan”, seed = 123, chains = 4, cores = 4)
# Obtains contrasts for the unadjusted model:
m1_simple %>%
  emmeans(~ intervention,
          epred = TRUE, re_formula = NULL) %>%
  contrast(method = “revpairwise”)
## Creates dataframe with 4k draws
m1_simpled <- m1_simple %>%
  emmeans(~ intervention,
          epred = TRUE, re_formula = NULL) %>%
  gather_emmeans_draws()
# Obtains contrasts for adjusted model:
m1 %>%
  emmeans(~ intervention | csofan,
          epred = TRUE) %>%
  contrast(method = “revpairwise”)
## Creates dataframe with 4k draws
```

```
m1d <- m1 %>%
  emmeans(~ intervention | csofan,
          epred = TRUE, re_formula = NULL) %>%
  gather_emmeans_draws()
## Splits in a list for calculations
m1ds <- m1d %>%
        select(-.chain,-.iteration,-.draw) %>%
        ungroup()%>%
        group_split(csofan)
# For unadjusted analysis, we extract the probabilities according to group and summarize:
prob_diff ← m1_simpled$.value[m1_simpled$intervention==1] - m1_simpled$.value[m1_simpled$intervention==0]
quantile(prob_diff,probs = c(0.025,0.50,0.975))
simple_or ← (m1_simpled$.value[m1_simpled$intervention==1]/(1-m1_simpled$.value[m1_simpled$intervention==1]))/
              (m1_simpled$.value[m1_simpled$intervention==0]/(1-m1_simpled$.value[m1_simpled$intervention==0]))
quantile(simple_or,probs = c(0.025, 0.50, 0.975))
sum(simple_or > 1)/length(simple_or) # Probability of harm
sum(between(simple_or, 1/1.1, 1.1))/length(simple_or) # Percentage of the posterior in ROPE
# For adjusted analysis, we manually calculated the values at each value of baseline SOFA and summarize. Only the first level (csofan == 0) is shown for clarity.
sofa0 <- m1ds[[1]]
or0<-(sofa0$.value[sofa0$intervention==1]/(1-sofa0$.value[sofa0$intervention==1]))/
  (sofa0$.value[sofa0$intervention==0]/(1-sofa0$.value[sofa0$intervention==0]))
quantile(or0,probs=c(0.025,0.50,0.975)) #Overall summary
sum(or0>1)/length(or0) # Probability of harm
sum(between(or0,1/1.1,1.1))/length(or0) # Percentage of posterior inside ROPE
#### END
```

Code for secondary analysis

```
#### START
#Set priors
nprior2 <- c(prior(normal(0, 0.355), class = "b"),
             prior("student_t(2.5, 0 , 10)", class = "b", dpar="phi"),
             prior("student_t(2.5, 0 , 10)", class = "b", dpar="zi"))
# Runs adjusted and unadjusted models
mdficu<-brm(bf(dficu | trials(28) ~ csofan*intervention,
              phi ~ csofan*intervention,
              zi ~ csofan*intervention),
            prior = nprior2,
            family = "zero_inflated_beta_binomial",
            chains = 4, cores = 4,
            backend="cmdstan",seed=123,data=maindata)

mdficu_simple<-brm(bf(dficu | trials(28) ~ intervention,
              phi ~ intervention,
              zi ~ intervention),
            prior = nprior2,
            family = "zero_inflated_beta_binomial",
            chains = 4, cores = 4,
            backend="cmdstan",seed=123,data=maindata)
```

```
# Extracts predictions for unadjusted model and summarizes results:
mdficu_simple %>%
  emmeans(~ intervention,
        epred = TRUE) %>%
  contrast(method = "revpairwise")
days_diff<-mdficu_simpled$.value[mdficu_simpled$intervention==1] - mdficu_simpled$.value[mdficu_simpled$intervention==0]
quantile(days_diff,probs=c(0.025,0.50,0.975))
sum(days_diff<0)/length(days_diff) # Probability of harm
sum(between(days_diff,-1,1))/length(days_diff) # Percentage of the posterior in ROPE
# Obtains contrasts for adjusted models, extracts and summarises posteriors from contrasts in adjusted model:
mdficu %>%
  emmeans(~ intervention | csofan,
        epred = TRUE) %>%
  contrast(method = "revpairwise")
mdficu_samples <- mdficu %>%
  emmeans(~ intervention | csofan,
        epred = TRUE) %>%
  contrast(method = "revpairwise") %>%
  gather_emmeans_draws()
mdficu_samples %>%
  select(-.chain,-.iteration,-.draw) %>%
  group_by(csofan) %>%
  summarise(m = sum(.value < 0)/n(),
          y = sum(between(.value,-1,1))/n())
#### END
```

rd deviation obtained from the results.

Table 1S - Results for cumulative logit model for organ dysfunction

	Odds ratio	Probability of harm*
Neurological SOFA in Day 3 according to baseline		
nSOFA 0	0.86 (0.55 - 1.20)	0.20
nSOFA 1	0.92 (0.49 - 1.48)	0.37
nSOFA 2	1.17 (0.62 - 1.94)	0.72
nSOFA 3	1.29 (0.75 - 1.94)	0.86
nSOFA 4	1.71 (0.98 - 2.68)	0.98
Hemodynamic SOFA in Day 3 according to baseline cardiovascular SOFA		
hSOFA 0	0.96 (0.67 - 1.36))	0.41
hSOFA 1	1.35 (0.73 - 2.48))	0.83
hSOFA 2	0.96 (0.45 - 2.02)	0.46
hSOFA 3	1.34 (0.78 - 2.37)	0.85
hSOFA 4	1.12 (0.71 - 1.74)	0.68
Renal SOFA in Day 3 according to baseline renal SOFA		
rSOFA 0	0.94 (0.63 - 1.37)	0.36
rSOFA 1	1.33 (0.80 - 2.21)	0.87
rSOFA 2	1.55 (0.84 - 3.05)	0.92
rSOFA 3	0.92 (0.44 - 1.97))	0.41
rSOFA 4	1.14 (0.52 - 2.42)	0.63

SOFA - Sequential Organ Failure Assessment Score. * Probability odds ratio > 1.0 for a transition to a higher state in the ordinal endpoint.

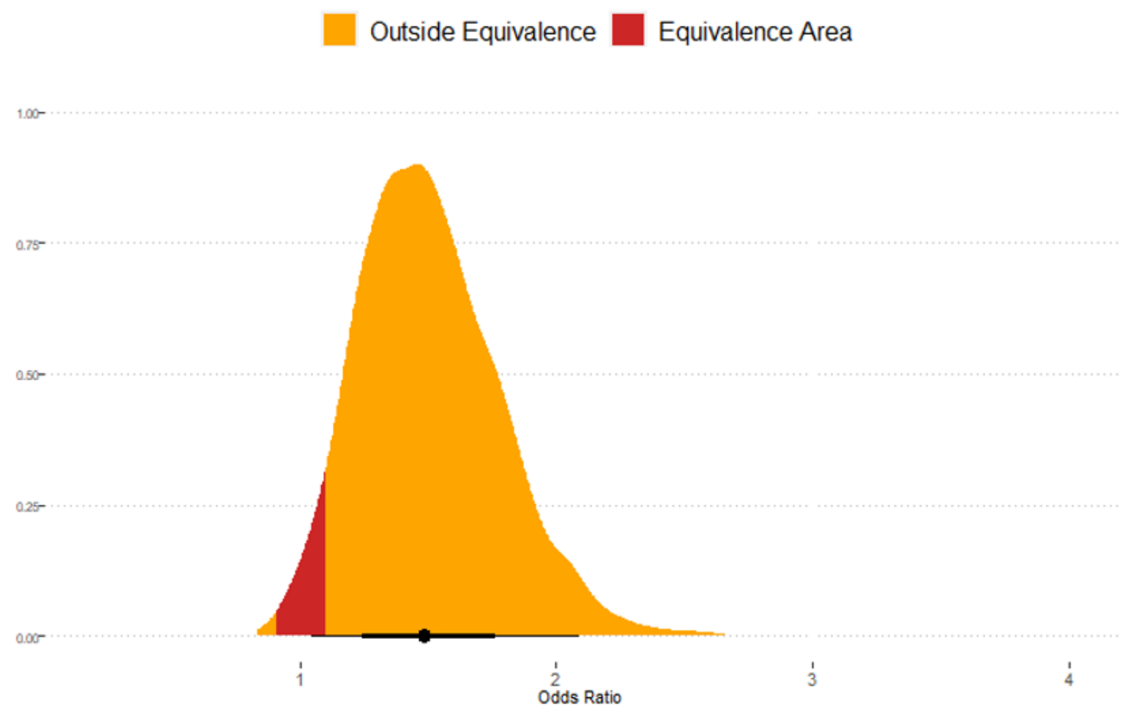

Figure 1S - Posterior odds ratio for mortality for the primary analysis with only intervention and random intercept for site as predictors.

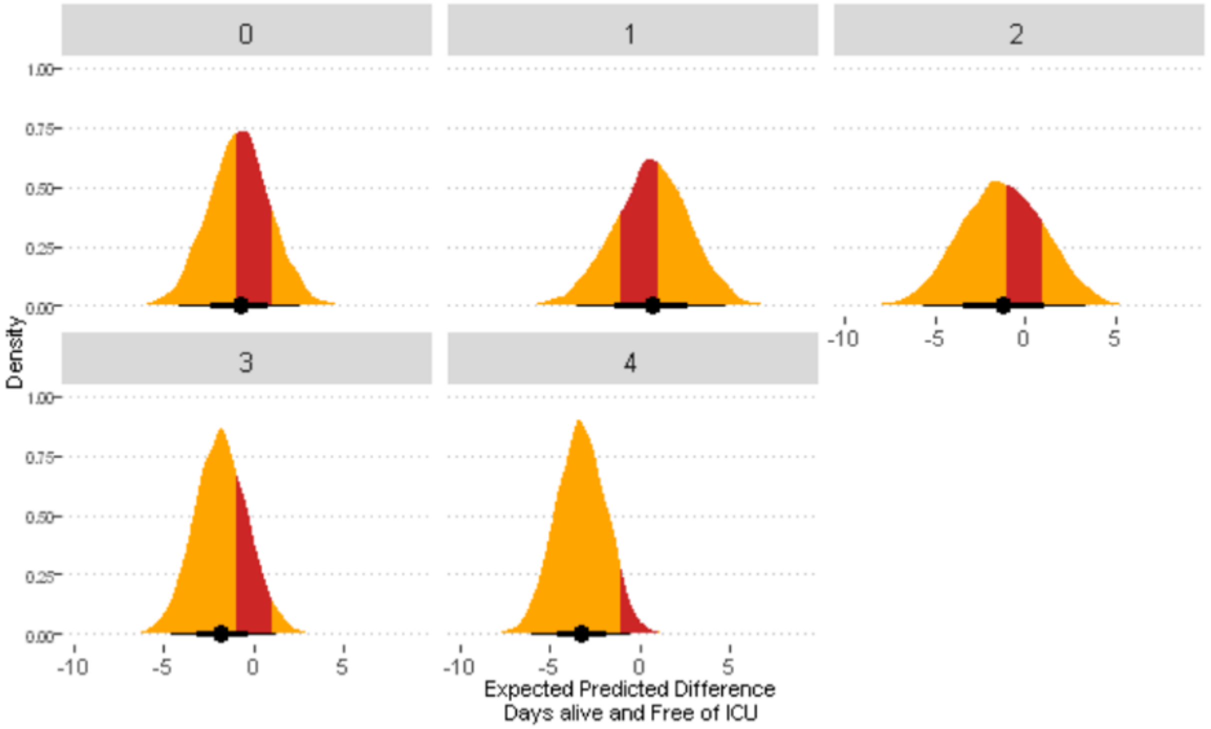

Figure 2S - Difference between expected posterior predicted number of days alive and free of intensive care unit according to baseline neurological Sequential Organ Failure Assessment score component and intervention. Region of equivalence (1 day) is shown in darker color.

ICU - intensive care unit.

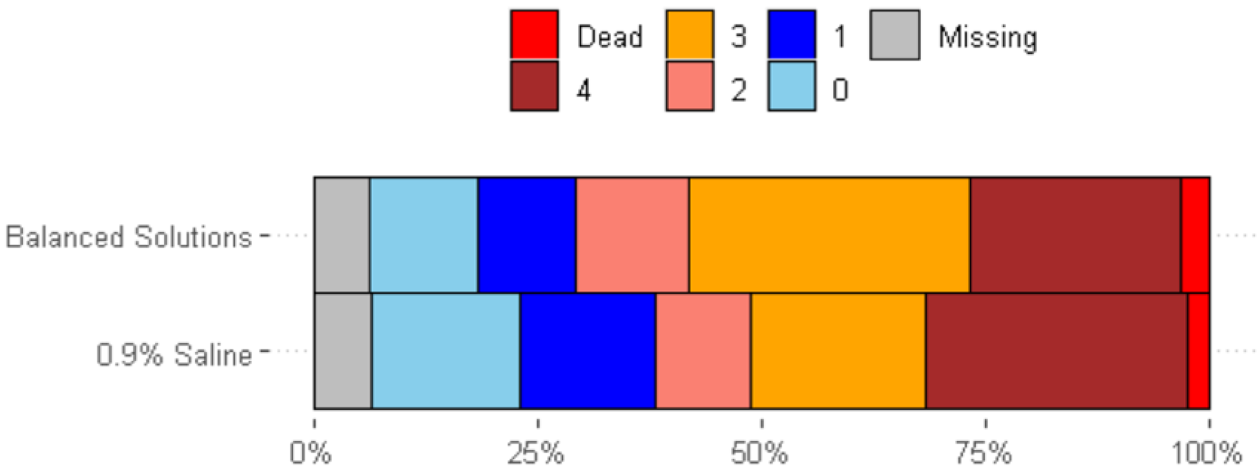

Figure 3S - Distribution of neurological Sequential Organ Failure Assessment score at Day 3, according to group.

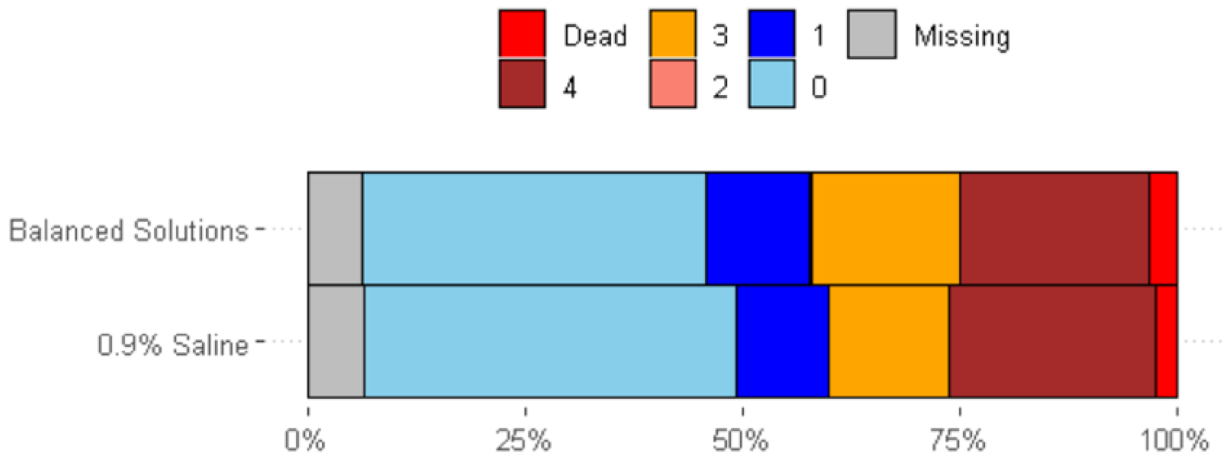

Figure 4S - Distribution of cardiovascular Sequential Organ Failure Assessment score at Day 3, according to group.

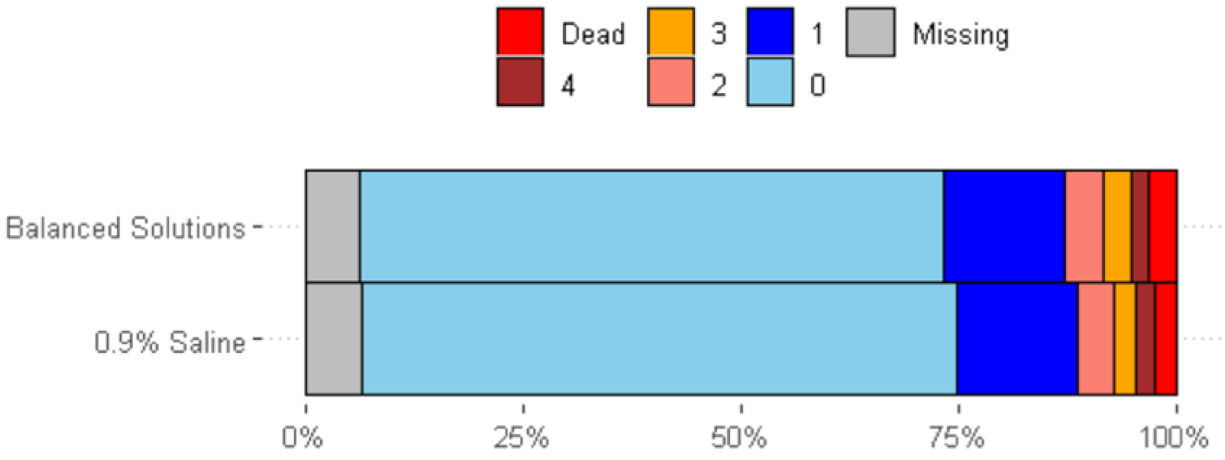

Figure 5S - Distribution of renal Sequential Organ Failure Assessment score at Day 3, according to group.
